# Correction: Kwon et al. The Effect of Integrative Treatment on Improving Functional Level in Stroke Patients: A Retrospective Chart Review. *Healthcare* 2025, *13*, 1452

**DOI:** 10.3390/healthcare13202582

**Published:** 2025-10-14

**Authors:** Daegil Kwon, Sun Hee Ahn, Hyun Jung Jung

**Affiliations:** 1 Comprehensive and Integrative Medicine Institute, Daegu 42473, Republic of Korea; cateyesn@naver.com (D.K.); ssuny4363@naver.com (S.H.A.); 2 Department of Diagnostics, College of Korean Medicine, Daegu Haany University, Daegu 41072, Republic of Korea

## Removal of an Author

Ki-cheul Shon was included as an author in the original publication [1] without his explicit consent for submission. At his request, his name has been removed from the list of authors. The corrected Author Contributions statement appears here. 

**Author Contributions:** Conceptualization, D.K. and H.J.J.; methodology, D.K. and H.J.J.; validation, S.H.A. and H.J.J.; formal analysis, D.K.; investigation, D.K., S.H.A., and H.J.J.; resources, S.H.A. and H.J.J.; data curation, S.H.A. and H.J.J.; writing—original draft preparation, D.K. and H.J.J.; writing—review and editing, D.K. and H.J.J.; visualization, D.K.; project administration, H.J.J. All authors have read and agreed to the published version of the manuscript.

## Affiliation Correction

In Affiliation 1, the city name in the address was misspelled as “Deagu”. This has been corrected to “Daegu”.

The authors state that the scientific conclusions are unaffected. This correction was approved by the Academic Editor. The original publication has also been updated.
